# Challenges to Exploring the Patient Perspective in Palliative Care Conversations: A Qualitative Study Among Chronic Obstructive Pulmonary Disease and Chronic Heart Failure Patients and Their Health Care Professionals

**DOI:** 10.1089/pmr.2023.0071

**Published:** 2024-04-05

**Authors:** Annet Olde Wolsink-van Harlingen, Leontine Groen-van de Ven, Kris Vissers, Jeroen Hasselaar, Jan Jukema, Madeleen Uitdehaag

**Affiliations:** ^1^ Research Group Smart Health, Saxion University of Applied Sciences, Deventer/Enschede, the Netherlands.; ^2^ Department of Anesthesiology, Pain and Palliative Medicine, Radboudumc University Medical Center, Nijmegen, the Netherlands.; ^3^ Research Group Living Well with Dementia, Windesheim University of Applied Sciences, Zwolle, the Netherlands.; ^4^ Nivel Netherlands Institute for Health Services Research, Utrecht, the Netherlands.

**Keywords:** challenges, chronic heart failure, chronic obstructive pulmonary disease, palliative care, patient perspective, person-centered communication

## Abstract

**Objectives:**

The aim of this study was to reveal the challenges faced in exploring the patient's perspective as experienced by patients with chronic obstructive pulmonary disease or chronic heart failure and their health care professionals (HCPs), including the circumstances under which these challenges are experienced during palliative care conversations.

**Methods:**

This is a qualitative, explorative study in the Netherlands using purposive sampling to create diversity in demographic variables of both patients and HCPs. Semistructured interviews with 12 patients and 7 HCPs were carried out with the use of topic lists. All interviews were audiorecorded, verbatim transcribed, and thematically analyzed.

**Results:**

Patients find it challenging to express their wishes, preferences, and boundaries and say what is really preoccupying them, especially when they do not feel a good connection with their HCP. HCPs find it challenging to get to know the patient and discuss the patient's perspective particularly when patients are not proactive, open or realistic, or unable to understand or recall information.

**Conclusions:**

Patients and HCPs seem to share the same aim: patients want to be known and understood and HCPs want to know and understand the patient as a unique individual. At the same time, they seem unable to personalize their conversations. To move beyond this impasse patients and HCPs need to take steps and be empowered to do so.

## Introduction

Chronic Obstructive Pulmonary Disease (COPD) and chronic heart failure (CHF) are two chronic life-limiting diseases both due to organ failure with similar symptom manifestation, disease trajectories, and unpredictable prognosis.^1–3^ The characteristics of both diseases make them eligible for palliative care (PC), also early in the disease trajectories to help improve quality of life and care.^4–6^ However, early integration of PC is challenging when patients' perceive their disease as chronic rather than life limiting and when there is a misconception of PC as synonym for end-of-life care. Another challenge is the difficult prognostication of both diseases affecting discussions of goals of care and advanced care planning.^7–9^

The same disease characteristics lead to complex PC needs, which require a person-centered approach.^10,11^ Person-centered PC should be based on partnership, ensuring that patients are enabled to participate in care and that decisions are respectful of and responsive to their values, needs, and preferences.^12,13^ Health care professionals (HCPs) can enable patients to participate in care and facilitate optimal information exchange, including the exploration of the patient perspective, by the use of person-centered communication.^14^ However, not every patient is able or willing to express their personal perspective^15–20^ and not every HCP is able or willing to explore this perspective during conversations.^21–24^ Patients find it difficult to talk about nonphysical dimensions and tend to avoid talking about emotional, social, or existential topics.^15–17^ They do not want to be bothersome, perceive time constraints, believe it is beyond the scope of their HCP, assume their perspective conflicts with the HCP's, and fear judgment.^15–20^ Patients with low health literacy, highly prevalent among patients with COPD and CHF, experience more barriers in expressing their perspective in conversations with HCPs.^18,25,26^

HCPs find it challenging to establish a relationship, get to know the person behind the patient, get information about quality of life, and develop a holistic perspective. They attribute these challenges to traditional practices, limited time, focus on physical aspects and treatments, and inability to adjust communication to patients with low health literacy.^21–23^

Thus, the exploration of patients' perspective in PC conversations with HCPs depends on the ability and willingness of both patients and HCPs and requires participation of patients in these conversations. However, participation depends on the knowledge, skills, attitude, and confidence of patients to participate in their care and on the relationship they have with their HCPs. Although patients can empower themselves the patient-provider relationship and person-centeredness can further enhance this process.^13^

The aim of this study is to reveal the challenges faced in exploring the patient's perspective as experienced by patients with COPD or CHF and their HCPs, including the circumstances under which these challenges are experienced during PC conversations. We need this knowledge to develop and implement a supportive toolbox for patients with COPD or CHF empowering them to express their personal perspective in PC conversations with HCPs.

To achieve this aim, this study focuses on the following questions: (1a) Which challenges are experienced by patients with COPD or CHF in expressing their personal perspective to HCPs? (1b) Under which circumstances are these challenges experienced by patients? (2a) Which challenges are experienced by HCPS in exploring the personal perspective of patients with COPD or CHF? (2b) Under which circumstances are these challenges experienced by HCPs?

## Methods

### Design

This study was part of a broader research and innovation project, called EMPATIE (EMpowerment of PATIEnts), covering two PC networks in the Eastern Netherlands. We started the project by exploring the challenges using two different research methods. First four co-creation sessions were held between October 2018 and February 2019.^27^ Second, semistructured face-to-face interviews with patients and HCPs were conducted between November 2018 and November 2019. This gave patients and HCPs unable or unwilling to participate in the cocreation sessions a voice in the project. This study presents the results of these semistructured interviews.

### Patients

Patients were recruited by HCPs who had already agreed to participate in the EMPATIE-project, during scheduled consultations. Interested patients were contacted face to face by one of the researchers who provided additional information and answered questions. Patients filled in an informed consent form and a demographic information sheet after giving verbal consent. Patients were then called to make an appointment for the interview. Second, the coordinator of a regional patient support organization sent an e-mail to eligible patients in their network. Interested patients were called by one of the researchers and provided with more information. After giving verbal informed consent, an appointment was made and the informed consent form and demographic information sheet were completed before the interview. Patients were purposively recruited to create a variety in diagnosis, gender, marital status, age, education, and time since diagnosis.

### Health care professionals

HCPs from hospitals, primary care practices, and homecare organizations were approached to participate in semistructured interviews. First, contact with HCPs was established through the coordinators of the two PC networks. Second, HCPs, who already agreed to participate in the EMPATIE-project, contributed to the recruitment of other HCPs within their personal network. Purposive sampling was used to create a variety in profession, setting, and years of experience. We planned to include at least one pulmonologist, one cardiologist, one advanced nurse practitioner (COPD), and one advanced nurse practitioner (CHF) working in a hospital, and one general practitioner (GP) and one practice nurse practitioner working in a primary care setting.

### Data collection

The semistructured interviews were carried out by researchers M.U., L.G.v.d.V., and A.O.W.v.H. Two topic lists were developed based upon the experience of the researchers, one for patients and one for HCPs (see Table 1). Patients were interviewed at home and HCPs at work.

**Table 1.** Topic Lists Patients and Health Care Professionals

Participant	Topics
Patients	- How patients experience conversations with HCPs. - What patients think a HCP needs to know about them to provide person-centered care. - What their HCPs know about them now. - What they want to tell or ask their HCP. - What challenges they experience in discussing topics or asking questions. - What they do when they experience these challenges. - In which situations they experience these challenges.
HCPs	- How HCPs experience their conversations with patients. - What HCPs find important in conversations with patients. - What challenges they experience when exploring the patient's perspective. - In which situations they encounter these challenges. - What HCPs do when they encounter these challenges.

HCP, health care professional.

### Data-analysis

All interviews were audiorecorded and transcribed verbatim. An inductive approach for developing themes was used. Transcripts were thematically analyzed by researcher A.O.W.v.H. using a six-phase process to familiarize the data and develop codes and themes.^28^ The transcripts of patients and HCPs were analyzed separately and systematically coded using ATLAS.ti 9.^28^ A.O.W.v.H. generated initial categories and themes from coded data. Initial codes, categories, and themes were further developed and reviewed through multiple individual discussions between A.O.W.-v.H. and researchers, M.U., J.J., and L.G.-v.d.V. Discussions between all participating researchers contributed to refining, defining, and naming of the themes. There was no threshold or limitation for including themes.

### Ethics

The Clinical Research Involving Human Subjects Act was not applicable to this study as declared by the Clinical Ethics Committee, Twente. Good clinical practice guidelines were applied to the informed consent procedure of participants.

## Results

### Participants

Twelve patients and seven HCPs, participated in this study. Four patient interviews, one with a COPD patient and three with CHF patients, were performed in the presence of one responsible informal caregiver. Five HCPs were working in a hospital setting and two in a primary care setting. Demographic information of patients is presented in Table 2 and of participating HCPs in Table 3. Interviews with patients had an average duration of 60 minutes and with HCPs 50 minutes.

**Table 2.** Demographic Information of Participating Patients

Characteristics	Patients (***n*** = 12)
Disease patient	
COPD	2
CHF	7
COPD and CHF	3
Gender	
Male	5
Female	7
Marital status	
Married/partner	9
Partner not living together	1
Widowed	2
Age	
40–50	2
61–70	1
71–80	4
81–90	5
Level of education^a^	
Low	2
Medium	9
High	1
Years since diagnosis	
<1 Year	3
2–5 Years	1
6–10 Years	3
>10 Years	5

^a^ Level of education High = college or university, Medium = secondary school, Low = vocational training.

CHF, chronic heart failure; COPD, chronic obstructive pulmonary disease.

**Table 3.** Demographic Information of Participating HCPs

Characteristics	HCP (***n*** = 7)
Profession	
GP	1
Practice nurse practitioner	1
Medical specialists	
Pulmonologist	1
Cardiologist	1
Advanced nurse practitioner	
COPD	1
CHF	1
PC	1
Setting	
Primary	2
Secondary	5
Gender	
Female	6
Male	1
Age	
31–40	1
51–60	5
61–70	1
Years of experience in their profession	
6–10	2
>10	4
Missing	1
Average number of COPD or CHF patients per month	
≤50	3
>50	3
Missing	1

GP, general practitioner; HCPs, Health care Professionals; PC, palliative care.

### Results patients

Patients reported different settings for their conversations with HCPs, such as during acute hospital admissions or long-term stays in a hospital, during medical tests or scheduled appointments in the outpatient clinic, and at home. Patients had positive and negative experiences of expressing their perspective during conversations with HCPs, which resulted in the formulation of two main challenges.

#### Challenge 1: Making my wishes, preferences, and boundaries known

This challenge relates to what patients value in their life, health, and care. The wishes relate to the desired improvement of physical functioning and abilities and the continuation of personal and social activities important to them. The preferences and boundaries relate to health care. Patients want to receive tailored care. They want to be heard in their experiential expertise, receive care according to their preferences, and receive clear explanations in layman's language. Patients also expressed two boundaries. They do not want HCPs to impose something on them and do not want contact on a personal level with every HCP (see Table 4).

**Table 4.** Challenges Experienced by Patients

Theme	Category	Code	Quotation
Making my wishes, preferences, and boundaries known	I want to physically function better	I want to feel better physically	“I'm particularly short of breath in the morning. I always ask for a bucket of air.” *Female, aged 82, widow, COPD and CHF*
		I want to be able to do more physically	“But cleaning the windows and hoovering everything, I can't do that yet. I want to get that back again. Yes, I do want to be able to do my normal things again.” *Female, aged 48, married, CHF*
	I want to be able to do things that are important to me	I want to be able to do fun things independently	“…..I always enjoy walking; I've always done that. But I can't go so far anymore.” *Male, aged 82, married, CHF*
		I want to be able to do nice things with my loved ones	“I still want to do so many nice things. With the children and with each other.” *Female, aged 69, married, COPD*
	I want to receive care that is right for me	I want to be heard for my knowledge gained through experience	“I've now got a regular cardiologist again; I've seen them once already. But when I saw them they got so carried away my husband said: “Calm down now!” She grabbed a folder and said: “Now, we have to increase this to the strongest dose. Then my husband said “Whoa - not so fast, I've got a story about that.” *Female, aged 72, married, CHF*
		I want to get care when I want it and from whom I want it	“At the hospital in [place] you ended up in a room on your own and they closed the door at night and I felt like I was in a prison and when the door was open then it was so busy in the corridor and uh, (sigh) there you were then. I sat waiting until the next day came. I thought it was terrible there.” *Female, aged 74, widow, CHF*
		I want a clear explanation in normal language	“I find the cardiologist quite accessible but they use loads more difficult words. It's easier to talk to the nurse.” *Male, aged 49, married, CHF*
		I do not want my HCP to impose something on me	“I have been able to put it [heart rehabilitation] off for three years, but they keep nagging on about it.” *Male, aged 80, living together, COPD and CHF*
		I do not feel the need for contact with a HCP on a personal level	“Everything I want to say, I can say to the nurses, to a couple of them anyway, not all of them. The younger ones are no good to me. But definitely with the pulmonary nurse and the oldest carer. They know me inside out. I don't need more than that.” *Female, aged 82, widow, COPD and CHF*
To say what is really preoccupying me	The influence the disease has on my life	That I cannot do as much physically because I do not feel good	“I used to always walk to the football pitch. That's a couple of kilometers outside the village, I can't manage that anymore and I think that's terrible.” *Male, aged 83, married, CHF*
		What I think and feel because I cannot do as much	“I need him [her husband] for everything. That's such a shame.” *Female, aged 69, married, COPD*
		How I experience the quality of my life	“I have often sat at the table howling that I can't carry on like this anymore. Then sometimes you call out: ‘If she has to live like this, then it's not worth it anymore’. *Female, aged 48, married, CHF*
	What comes to mind when I think about my future/end of life	That I want to know what I can expect in the future	“…whether I have any chance of going back to work again. What will I still be able to do in the near future? Will I become a vegetable? *Male, aged 49, married, CHF*
		That I have ambivalent feelings about the end of my life	“Because sometimes as far as I'm concerned, it's not worth it anymore. Then I think but there's no turning back. Then sometimes I think just let me carry on sleeping. But I don't want to go, I don't want to leave the children and I still want to do so much [gets emotional]. But I just can't manage it.” *Female, aged 69, married, COPD*
		What thoughts and worries I have	“Well, I wake up and I'm a burden to him [her husband].” *Female, aged 69, married, COPD*
	What my negative experiences are with my health care professional	That I blame my HCP because they have made mistakes	“I was not so happy with the new cardiologist. He was a nice bloke but he made a couple of mistakes.” *Female, aged 88, married, CHF*
		That my HCP does not do what is expected of them	“He (GP) hasn't called to find out how it actually went. I had expected that he would. That would have certainly been polite of him.” *Male, aged 48, married, CHF*
		I do not agree with the treatment prescribed by my health care professional	“I get given morphine but I asked for a sleeping pill. The doctor says I can have it [morphine] every other day. Well, I'm not happy about that. I want it every day. I disagree with this, because otherwise I'm very restless.” *Female, aged 69, married, COPD*
		That my HCP lacks empathy in their communication with me	“….we [cardiologists] have finished treating you, we [cardiologists] can't do anything more for you. I felt really anxious because I thought so, ‘finished with treatment and we can't do anything else for you because your heart is so bad’. I thought this is it then, the end. That's what it seemed like to me. That's how I understood it and I was totally out of sorts…You do take account of it but never really think what it actually means.” *Female, aged 72, married, CHF*
		That the test results mean more to my HCP than how I feel	“We went to the doctor's and she said that everything was good. The heart film is good, blood is good, blood pressure is good, everything is good. And I said but I don't feel good.” *Female, aged 88, married, CHF*

#### Challenge 2: To say what is really preoccupying me

Patients indicate that it can be hard to express the impact the disease has on their lives. They find it difficult to share what comes to mind when they think about their final stage of life. Although they want to know what to expect, they also have ambivalent feelings about it. They are aware of the relevance of talking about their wishes, thoughts, and concerns about future care, but find it hard to do so. Finally, they also experience difficulties in talking about negative experiences with HCPs regarding communication, decision making, or the provision of care (see Table 4).

These two challenges are encountered under two identified circumstances.

#### Circumstance 1: When I do not have a bond with my HCP

Reasons mentioned by patients are HCPs having limited time, discontinuity of HCPs, or limited contact moments in the care process. Several patients expressed frequent changes in HCPs. When patients receive their care in a hospital, their GP is often not involved. Patients may not feel a connection with their HCP because they do not experience a click or because the HCP acts aloof. Finally, not feeling equal to the HCP due to experiencing hierarchy or feeling patronized can contribute to patients not feeling they have established a relationship with their HCP (see Table 5).

**Table 5.** Circumstances Under Which Challenges Are Encountered by Patients

Theme	Category	Code	Quotations
I do not have a bond with my health care professional	I do not see or speak to my HCP very often	The doctor does not have much time	“Then I got a bit of a speedy Gonzales, it was all a bit too quick for me. He did everything so quickly. I did trust him, but you just didn't really have any contact.” *Female, aged 72, married, COPD and CHF*
		I only see the doctor/nurse once or twice a year	“My first hospital admittance was this year while I've been seeing him for five years now. In those five years, you see him twice a year at the most, so you don't really develop a bond.” *Female, aged 72, married, COPD and CHF*
		The GP is not in the picture	“The internist is my main practitioner. I have little contact with my GP.” *Female, aged 74, widow, CHF*
		I keep getting different doctors (GP/cardiologist)	“We've had so many changes in recent years. Also with cardiologists, you never really have a bond with them, with those people. They leave too quickly.” *Female, aged 88, married, CHF*
	I do not feel any connection with my health care professional	I do not experience a ‘click’	“To be honest, I didn't really like her [heart failure nurse] that much. And then you are less inclined to say much.” *Female, aged 82, widow, COPD and CHF*
		I think that the HCP acts detached	“We could talk easily with the first cardiologist. About symptoms or anything else. But when they are so stand-offish. And that's the case now.” *Female, aged 88, married, CHF*
	I do not feel like an equal to my health care professional	I get the feeling that my HCP is above me	“Because this GP shows empathy. The previous GP tended to look down on you. They would briefly tell you what you had to do.” *Male, aged 80, living together, COPD and CHF*
		I get the impression that the HCP does not regard me as an equal	“They [two nurses] had a whole story about all that needed to be done. And, you know what? And he [her husband] says: “I can still see your face; completely speechless you were.” As if I was some kind of stupid old person. I really felt that strongly then, that I was being treated as inferior. *Female, aged 88, married, CHF*
I cannot have a good conversation with my health professional	I cannot make good contact with my health professional	We do not make eye contact	“They need to listen to me properly and what really irritates me is that they have a screen in front of them. If I only have 10 minutes and they sit looking at their screen for almost 10 minutes, then I don't feel like I'm being taken seriously. I want us to look at one another and talk to each other. That he can also see me, because when you look at someone you make contact.” *Female, aged 72, married, COPD and CHF*
		The conversation is completely one-sided	“He [GP] lets people talk a bit when you are there. Then he sits and looks at you for a bit.” *Female, aged 88, married, CHF*
	I do not feel any depth to our conversations	Our contact is superficial	Interviewer: “Which health professional do you know the best?” Respondent: “No idea. It's all so superficial.” *Female, aged 82, widow, COPD and CHF*
		We only talk about physical/medical matters	“We always discuss health and what can be done about it. Always the medical aspects.” *Female, aged 82, widow, COPD and CHF*
	I do not give or ask for information myself	It does not seem useful to me to share certain information with my health professional	“Ooh, but we don't even talk about that either [physical deterioration]. No, they [healthcare professionals] can't do anything about that anyway.” *Female, aged 72, married, COPD*
		I do not feel important enough to ask for attention for myself	“Ach, even if I do say what I think, I'm a nobody who's going to become nothing, so just forget it.” *Female, aged 82, widow, COPD and CHF*
		I do not know what I can say and/or ask	“Well yes, I never really have anything to discuss. Only if I feel something or whatever. And when it's going well, what do I say then, I'm fine thanks?” *Female, aged 76, married, CHF*

#### Circumstance 2: When I cannot have a good conversation with my HCP

This situation occurs when a patient fails to have in-depth contact with a HCP, when they experience insufficient trust or willingness in conversations, when the patient does not provide or ask for information themselves. Patients would like to have eye contact and a bidirectional conversation. They also wish for deeper contact with their HCP and the ability to talk about other topics than just physical and medical issues. The fact that a patient does not share or ask for information can be due to the fact that the patient thinks there is no use in doing so. This factor plays a role when patients believe HCPs cannot say or do anything about their situation, when they think they are not worthy of asking attention for themselves or when they do not know what to say or ask when everything seems to go well (see Table 4).

### Results HCPs

HCPs mainly talked about scheduled conversations in consultation rooms or the patient's home. HCPs talked less about conversations during hospital admissions, except the PC nurse. HCPs expressed several challenges to exploring the patient's perspective which, according to them, can lead to the delivery of suboptimal care. This resulted in the identification of the following two challenges.

#### Challenge 1: Getting to know the patient better

HCPs find it difficult to have in-depth contact with a patient because they cannot find a connection with them. Furthermore, HCPs find it difficult to explore what is going on in the mind of patients. It is difficult to get to the core and explore the readiness of patients to talk about end of life. HCPs find it challenging to discuss sensitive or nonmedical topics like emotions, sexuality, concerns about independence, prognosis, or the future, when patients also find it difficult to talk about these subjects (see Table 6).

**Table 6.** Challenges Experienced by Health Care Professionals

Theme	Category	Code	Quotation
Getting to know the patient better	I find it difficult to make good contact with patients	Difficult to find a way to engage	“I sometimes find it difficult to start a conversation with these people [patients with COPD]. To remove the barrier they have, a sort of reservation, in order to be able to start a chat.” *Advanced Nurse Practitioner Palliative Care, female, aged 52*
		Difficult to build rapport	“Sometimes you just can't make contact, be let in. And then they leave and you just think phew, that sort of feeling. Sometimes you pull harder without knowing it and then after they have gone I'd think: I didn't go about that the right way. I've been trying too hard to build rapport when someone is just not motivated.” *Advanced Nurse Practitioner COPD, female, aged 51*
	I find it difficult to work out what is going on inside the patient's mind	Difficult to get to the heart of the matter	“To try and find where the crux is for patients. To be able to offer them the tools to deal with it better. That's always a challenge with every patient, finding the best way to get it out of them. *Advanced Nurse Practitioner COPD, female, aged 51*
		Difficult to ascertain whether patients want to talk about end of life	“Because the course of the disease can be so different and is so difficult to predict. When is the right time to have the [bad news] conversation, that is really difficult? Sometimes we think now's a good moment, but then the patient turns out not to be ready for it.” *Advanced Nurse Practitioner CHF, female, aged 38*
	I find it difficult to talk about certain subjects when the patient does not want to or cannot	The patient finds it difficult to talk about nonmedical/physical subjects (independence, prognosis)	“Well, what you don't get to hear, I think, especially as a doctor, are the things going on in someone's mind. Concerns about remaining independent, worries about life expectancy.” *Pulmonologist, female, aged 52*
		The patient finds it difficult to talk about sensitive matters (sexuality, emotions, future)	“If you start talking about the future, they quickly find that an uncomfortable subject. They more or less close their eyes to it. All they really want to hear from me is that everything is fine. *Cardiologist, female, aged 61*
Making the patient's perspective a topic of discussion	I do not give the patient an opportunity to contribute	I have little time for conversation	“Of course, our time is limited.” *Cardiologist, female, aged 61*
		My own conversation agenda takes up a lot of time	… “we have to quickly run through the medical-technical part, we first need to look at any heart complaints, then we need the blood pressure, listen to the lungs, see if there is any fluid in the legs, look at the ECG, view the blood results, look at data from the ICD or pacemaker, check medication and then we are more or less finished, but by then the time is often up.” *Cardiologist, female, aged 61*
		I talk a lot myself	“Because doctors are terrible for talking and the patient hardly gets any time to say anything.” *Cardiologist, female, aged 61*
	I find it difficult to talk openly about some subjects (fear, strain, life questions) or finding the right moment to broach them	I do not know the patient well	“People don't talk about these subjects easily themselves, such as fear, strain, life questions. That's highly personal of course, not just something you casually mention. So, you often only ask someone about these things after you know them a bit better.” *Advanced Nurse Practitioner COPD, female aged 51*
		I cannot find an opening to start talking about end of life	“….those are the conversations, yes, that are really difficult at times. After a hospital admission due to heart failure, they often perk up a lot and think “I'm back again!” Then they are absolutely in no mood for that conversation. Finding the right moment to talk about it is always a big challenge.” *Advanced Nurse Practitioner CHF, female, aged 38*
		I (no longer) dare to broach difficult subjects (prognosis, sexuality, lifestyle).	“In the sense that I think that I've sometimes made mistakes by deciding to say something and then they end up still going strong six months later. I think I've got that completely wrong a couple of times and so I wouldn't say that so quickly anymore.” *Advanced Nurse Practitioner CHF, female, aged 38*

#### Challenge 2: Making the patient's perspective a topic of discussion

HCPs experience no space for integrating the personal perspective of patients due to lack of time, their own extensive agenda for the consultation, or because they speak a lot themselves. Another reason for this challenge is that HCPs find it difficult to talk about sensitive topics like sexuality, social isolation, prognosis, or end of life. They find it challenging when they do not know the patient or when they have negative experiences discussing these topics (see Table 6).

These two challenges of HCPs are experienced under two identified circumstances.

#### Circumstance 1: When the patient does not behave the way I would expect

HCPs seem to hope for patients who are proactive, realistic, and open and find it difficult when they act otherwise. They gave examples about patients who arrived unprepared, do not take initiative, present their symptoms or problems insufficiently, do not want to hear or face the consequences of their disease or, are unable or unwilling to open up (see Table 7).

**Table 7.** Circumstances Under Which Challenges Are Encountered by Health Care Professionals

Theme	Category	Code	Quotation
The patient does not behave the way I would expect	The patient is not proactive	The patient comes to the appointment unprepared	“…. you can count them on one hand, the people that come to a consultation well prepared.” *Advanced Nurse Practitioner COPD, female aged 51*
		The patient does not take initiative/is not assertive	“You forget just how many people come here and sit-down thinking “Well, get on with it then doctor or nurse.” That I think yes, but it is an interactive consultation.” *Advanced Nurse Practitioner CHF, female, aged 38*
	The patient is not realistic	The patient presents their symptoms/problems insufficiently	“Many people have the tendency to trivialise their complaints. Despite the fact they have come to see that person specifically to discuss their complaints. Perhaps from a sort of basic feeling that you want to present yourself as well as possible. *Advanced Nurse Practitioner COPD, female aged 51*
		The patient does not want to hear/know the consequences of the disease	“Because not everyone wants to know where they stand. Some people just don't want to know or they simply look away or don't hear it when you say that you are concerned and I don't think you've got long to live.” *Advanced Nurse Practitioner CHF, female, aged 38*
	The patient is not open	The patient finds it difficult	“Sometimes you have consultation hours that drain your energy. That you've had to work hard to extract all the information.” *Advanced Nurse Practitioner COPD, female, aged 51*
		The patient still does not want to know	“There are people who are naturally closed, making it difficult to know whether someone is ready for such a conversation (end of life).” *Advanced Nurse Practitioner COPD, female, aged 51*
		The patient has no click with me	“You don't have much influence on whether the click is there or not, but someone who feels a click, will find it easier to ask you or tell you something, than when there's no click.” *Pulmonologist, female, aged 52*
The patient does not understand the conversation or information	The patient does not possess the ability	The patient has insufficient cognitive ability	“Sometimes someone is cognitively limited. They simply don't possess the cognition to cope well with their disease, or even to cope with their life. It is a case of continually seeking the level of the patient and communicating on that level. That you talk on the same level and don't use overly complicated words. That is different for each individual patient.” *Advanced Nurse Practitioner COPD, female, aged 51*
		The patient cannot remember all the conversation/information	“When I explain the euthanasia procedure then I do it slowly, step for step. People say, great, crystal-clear doctor, I agree, but what they still remember the next day, I just don't know.” *General Practitioner, male, aged 54*
	The patient has insufficient knowledge and insight	The patient has insufficient knowledge and insight into their disease and treatment	“I think that I leave it a while when people don't understand it and perhaps that's not always a good thing to do. So that's still a challenge.” *Advanced Nurse Practitioner CHF, female, aged 38*
		The patient is not good at interpreting information in the media	“Yes, of course the media often has a really big influence. When, for example, there is a new asthma medicine available and then COPD patients come and ask, “Is that not something I could use?” And it is totally unsuitable for COPD.” *Pulmonologist, female, aged 51*

#### Circumstance 2: When the patient does not understand the conversation or information

HCPs also find it difficult when they experience patients' inability to understand the conversation or information. This can be caused by limited cognitive and communication capacities or recall problems due to low health-literacy, high age, or disease severity. It can also be caused by insufficient knowledge and insight in the disease and treatment or because patients have their own assumptions and beliefs about medical tests and interventions (see Table 7).

## Discussion and Conclusion

### Summary

This explorative qualitative study revealed challenges to exploring the patients' perspective experienced by patients with COPD or CHF and their HCPs, including the circumstances under which these challenges are experienced. Patients find it challenging to express their wishes, preferences, and boundaries relating to what they value in life, health, and care and saying what is really preoccupying them. HCPs find it challenging to get to know the patient well and discuss the patient's perspective. Patients experience these challenges when they do not feel a good connection or when they are unable to have a good conversation with their HCP. HCPs experience these challenges when patients are not proactive, open or realistic, or are unable to understand or recall information.

### The impasse

These challenges and circumstances reflect what has been described in other studies.^15–23^ Particularly challenging is the timing and discussion of prognosis and end of life due to the unpredictable course and prognosis of both diseases.^29–32^ Limited health literacy (LHL) can be an influencing factor as well.^26,33,34^ Both patients and HCPs find it hard to initiate these discussions and prefer the other to take the initiative.^32,35,36^ This may result in HCPs keeping their focus on physical and medical aspects of care instead of quality of life and preparing for the end of life.^31,35,37,38^

Ironically, patients and HCPs seem to share the same aim: patients want to be known and understood and HCPs want to know and understand the patient as unique individual. Patients find it difficult to be seen, heard, and understood as unique individuals. HCPs find it hard to make a connection and explore the unique perspective of individual patients. Despite the congruent preferences they do not easily succeed to personalize their conversations. The implication is that the patient's perspective is not explored and expressed in PC conversations. This impacts the HCP's understanding of the patient as a person and the development of a therapeutic relationship. The provision of person-centered PC then becomes more difficult or even impossible.

It is important to break out of this impasse. However, HCPs tend to prioritize their own agenda, focus on physical and medical subjects, and are unable to effectively communicate or connect with the patient.^21–24,39,40^ It is possible that HCPs have learned or are triggered by the system to use a certain format and style for conversations with patients.^23,40,41^ Under these circumstances it is challenging for patients to make themselves known as a unique individual. Patients are not used to preparing for conversations, are sometimes unable or unwilling to talk about certain subjects, or are unable to understand or recall information.^15,16,18,20,36,37,42,43^ Under these circumstances it is challenging for HCPs to get to know the person behind the patient and provide tailored care.

Although patients and HCPs mentioned circumstances relating to their own competencies and attitudes, they both tended to focus on the other in explaining why they experience these challenges.^17,31,32,35,36,38,44^ However, moving beyond this impasse requires different behavior of both patients and HCPs. Patients need to prepare for, and actively participate in conversations. However, such a role is not obvious to all patients^44^ and patients need a certain level of empowerment to be capable to engage in their health care.^13^ HCPs need to be aware that LHL influences patient empowerment, participation, and effectiveness of communication.^13,21,34,37,43^ They need to support patients in their active role by enhancing the relationship with the patient, inviting the patient to share their perspective, and by communicating in a sensitive manner.^44–46^ This seems especially challenging for medical specialists probably due to the limited time and their perceived role and responsibility.^20,21^

### Strengths and limitations

A strength of this study is that it simultaneously studied challenges and circumstances of both patients and HCPs, which made it possible to discover the impasse. Another strong point is that the themes were refined, defined, and named through multiple discussions with the participating researchers.

In four interviews three male partners and one adult daughter were present during data collection. Although we did not include their expressions in our analysis, their presence could have influenced patient disclosures.

### Conclusion

This study provides the insight that patients and HCPs seem to strive for the same thing: patients want to be known and understood and HCPs want to know and understand the patient as unique individual. At the same time, they seem unable to personalize conversations. Although patients and HCPs mentioned factors relating to themselves, they mainly tended to focus on the behavior of the other. Breaking this impasse requires both patients and professionals to take steps and both need to be empowered to do so. This could be realized with the use of (inter-) national evidence-based conversation tools for both patients and HCPs.

### Practical implications and research recommendations

Although the EMPATIE-project is focusing on the empowerment of patients, this study revealed that both patients and HCPs need to become aware of the relevance of integrating the patient's perspective into PC conversations. They both need to be empowered to change their behavior. It is important to study their behavior in more depth in a more diverse population during PC conversations. Also, further study is required to integrate the patient's perspective into PC conversations, including the required implementation strategies.

## Abbreviations Used

CHF: chronic heart failure
COPD: chronic obstructive pulmonary disease
EMPATIE: EMpowerment of PATIEnts
GP: general practitioner
HCP: health care professional
LHL: limited health literacy
PC: palliative care

## References

[1] Murray SA, Kendall M, Grant E, et al. Patterns of social, psychological, and spiritual decline toward the end of life in lung cancer and heart failure. J Pain Symptom Manage 2007;34(4):393–402; doi: 10.1016/j.jpainsymman.2006.12.009 17616334

[2] Giacomini M, DeJean D, Simeonov D, et al. Experiences of living and dying with COPD: A systematic review and synthesis of the qualitative empirical literature. Ont Health Technol Assess Ser 2012;12(13):1–47. PMC3384365 23074423

[3] Klindtworth K, Oster P, Hager K, et al. Living with and dying from advanced heart failure: Understanding the needs of older patients at the end of life. BMC Geriatr 2015;15:125; doi: 10.1186/s12877-015-0124-y 26470713 PMC4608315

[4] Fitzsimons D, Mullan D, Wilson JS, et al. The challenge of patients' unmet palliative care needs in the final stages of chronic illness. Palliat Med 2007;21(4):313–322; doi: 10.1177/0269216307077711 17656408

[5] Hauptman PJ, Havranek EP. Integrating palliative care into heart failure care. Arch Intern Med 2005;165(4):374–378; doi: 10.1001/archinte.165.4.374 15738365

[6] WHONFSDPCAoahwwin-rf-sdp-c. Available from: https://www.who.int/news-room/fact-sheets/detail/palliative-care [Last accessed: March 2023].

[7] Kavalieratos D, Mitchell EM, Carey TS, et al. “Not the ‘grim reaper service’”: An assessment of provider knowledge, attitudes, and perceptions regarding palliative care referral barriers in heart failure. J Am Heart Assoc 2014;3(1):e000544; doi: 10.1161/JAHA.113.000544 24385453 PMC3959712

[8] Hadler RA, Curtis BR, Ikejiani DZ, et al. “I'd Have to Basically Be on My Deathbed”: Heart failure patients' perceptions of and preferences for palliative care. J Palliat Med 2020;23(7):915–921; doi: 10.1089/jpm.2019.0451 31916910 PMC7249460

[9] Tavares N, Jarrett N, Wilkinson TMA, et al. Patient-centered discussions about disease progression, symptom, and treatment burden in chronic obstructive pulmonary disease could facilitate the integration of end-of-life discussions in the disease trajectory: Patient, clinician, and literature perspectives: A Multimethod Approach. J Palliat Med 2023;26(3):353–359; doi: 10.1089/jpm.2022.0028 36251863

[10] Janssen DJ, Franssen FM, Wouters EF, et al. Impaired health status and care dependency in patients with advanced COPD or chronic heart failure. Qual Life Res 2011;20(10):1679–1688; doi: 10.1007/s11136-011-9892-9 21442430 PMC3220822

[11] Siouta N, van Beek K, Preston N, et al. Towards integration of palliative care in patients with chronic heart failure and chronic obstructive pulmonary disease: A systematic literature review of European guidelines and pathways. BMC Palliat Care 2016;15:18; doi: 10.1186/s12904-016-0089-4 26872741 PMC4752742

[12] Committee on Quality of Health Care in America, Institute of Medicine. Crossing the Quality Chasm: A New Health System for the 21st Century. National Academies Press (US): Washington (DC); 2001.

[13] Hickmann E, Richter P, Schlieter H. All together now—Patient engagement, patient empowerment, and associated terms in personal healthcare. BMC Health Serv Res 2022;22 (1):1116; doi: 10.1186/s12913-022-08501-5 36056354 PMC9440506

[14] Epstein RM, Street RL Jr. Patient-Centered Communication in Cancer Care: Promoting Healing and Reducing Suffering. NIH Publication No. 07-6225. National Cancer Institute: Bethesda, MD; 2007.

[15] Lim C, Berry ABL, Hirsch T, et al. “It just seems outside my health”: How patients with chronic conditions perceive communication boundaries with providers. DIS (Des Interact Syst Conf) 2016;2016:1172–1184; doi: 10.1145/2901790.2901866 28804790 PMC5553690

[16] Joensson ABR, Guassora AD, Freil M, et al. What the doctor doesn't know: Discarded patient knowledge of older adults with multimorbidity. Chronic Illn 2020;16(3):212–225; doi: 10.1177/1742395318796173 30213205

[17] Barry CA, Bradley CP, Britten N, et al. Patients' unvoiced agendas in general practice consultations: Qualitative study. BMJ 2000;320(7244):1246–1250; doi: 10.1136/bmj.320.7244.1246 10797036 PMC27368

[18] Henselmans I, Heijmans M, Rademakers J, et al. Participation of chronic patients in medical consultations: Patients' perceived efficacy, barriers and interest in support. Health Expect 2015;18(6):2375–2388; doi: 10.1111/hex.12206 24813384 PMC5810699

[19] van Bruinessen IR, van Weel-Baumgarten EM, Gouw H, et al. Barriers and facilitators to effective communication experienced by patients with malignant lymphoma at all stages after diagnosis. Psychooncology 2013;22(12):2807–2814; doi: 10.1002/pon.3352 23897828

[20] Alders I, Henselmans I, Smits C, et al. Patient coaching in specialist consultations. Which patients are interested in a coach and what communication barriers do they perceive? Patient Educ Couns 2019;102(8):1520–1527; doi: 10.1016/j.pec.2019.03.011 30910403

[21] Roodbeen R, Vreke A, Boland G, et al. Communication and shared decision-making with patients with limited health literacy; helpful strategies, barriers and suggestions for improvement reported by hospital-based palliative care providers. PLoS One 2020;15(6):e0234926; doi: 10.1371/journal.pone.0234926 32559237 PMC7304585

[22] Schuttner L, Hockett Sherlock S, Simons CE, et al. My goals are not their goals: Barriers and facilitators to delivery of patient-centered care for patients with multimorbidity. J Gen Intern Med 2022;37(16):4189–4196; doi: 10.1007/s11606-022-07533-1 35606644 PMC9126696

[23] Moore L, Britten N, Lydahl D, et al. Barriers and facilitators to the implementation of person-centred care in different healthcare contexts. Scand J Caring Sci 2017;31(4):662–673; doi: 10.1111/scs.12376 27859459 PMC5724704

[24] Barry CA, Stevenson FA, Britten N, et al. Giving voice to the lifeworld. More humane, more effective medical care? A qualitative study of doctor-patient communication in general practice. Soc Sci Med 2001;53(4):487–505; doi: 10.1016/s0277-9536(00)00351-8 11459399

[25] Roberts NJ, Ghiassi R, Partridge MR. Health literacy in COPD. Int J Chron Obstruct Pulmon Dis 2008;3(4):499–507; doi: 10.2147/copd.s1088 19281068 PMC2650589

[26] Cajita MI, Cajita TR, Han HR. Health literacy and heart failure: A systematic review. J Cardiovasc Nurs 2016;31(2):121–130; doi: 10.1097/JCN.0000000000000229 25569150 PMC4577469

[27] van Harlingen AOW, van de Ven LG, Hasselaar J, et al. Developing a toolkit for patients with COPD or chronic heart failure and their informal caregivers to improve person-centredness in conversations with healthcare professionals: A Design Thinking approach. Patient Educ Couns 2022;105(11):3324–3330; doi: 10.1016/j.pec.2022.07.002 35843846

[28] Braun V, Clarke V. Thematic Analysis. A Practical Guide, Vol 3. Qualitative Research in Psychology, Vol 2. SAGE Publications Ltd.: London; 2022.

[29] Ngwenya N, Crang C, Farquhar M, et al. Communicating uncertainty: contrasting the communication experiences of patients with advanced COPD and incurable lung cancer. Fam Pract 2021;38(5):637–643; doi: 10.1093/fampra/cmab024 33871548 PMC8604275

[30] Cavanagh CE, Rosman L, Spatz ES, et al. Dying to know: Prognosis communication in heart failure. ESC Heart Fail 2020;7(6):3452–3463; doi: 10.1002/ehf2.12941 32969195 PMC7754721

[31] Hjelmfors L, Sandgren A, Strömberg A, et al. “I was told that I would not die from heart failure”: Patient perceptions of prognosis communication. Appl Nurs Res 2018;41:41–45; doi: 10.1016/j.apnr.2018.03.007 29853212

[32] Momen N, Hadfield P, Kuhn I, et al. Discussing an uncertain future: End-of-life care conversations in chronic obstructive pulmonary disease. A systematic literature review and narrative synthesis. Thorax 2012;67 (9):777–780; doi: 10.1136/thoraxjnl-2012-201835 22802331

[33] Nouri SS, Barnes DE, Volow AM, et al. Health literacy matters more than experience for advance care planning knowledge among older adults. J Am Geriatr Soc 2019;67(10):2151–2156; doi: 10.1111/jgs.16129 31424575 PMC6831086

[34] Otte R, Roodbeen R, Boland G, et al. Affective communication with patients with limited health literacy in the palliative phase of COPD or lung cancer: Analysis of video-recorded consultations in outpatient care. PLoS One 2022;17(2):e0263433; doi: 10.1371/journal.pone.0263433 35143534 PMC8830703

[35] Houben CH, Spruit MA, Schols JM, et al. Patient-clinician communication about end-of-life care in patients with advanced chronic organ failure during one year. J Pain Symptom Manage 2015;49(6):1109–1115; doi: 10.1016/j.jpainsymman.2014.12.008 25623920

[36] Tavares N, Hunt K, Jarrett N, et al. The preferences of patients with chronic obstructive pulmonary disease are to discuss palliative care plans with familiar respiratory clinicians, but to delay conversations until their condition deteriorates: A study guided by interpretative phenomenological analysis. Palliat Med 2020;34(10):1361–1373. (1477-030X (Electronic)) 32720555 10.1177/0269216320937981

[37] Noordman J, Schulze L, Roodbeen R, et al. Instrumental and affective communication with patients with limited health literacy in the palliative phase of cancer or COPD. BMC Palliat Care 2020;19(1):152; doi: 10.1186/s12904-020-00658-2 33028308 PMC7542099

[38] Tavares N, Jarrett N, Hunt K, et al. Palliative and end-of-life care conversations in COPD: A systematic literature review. ERJ Open Res 2017;3(2):00068-02016; doi: 10.1183/23120541.00068-2016 28462236 PMC5407435

[39] Kowalski CP, McQuillan DB, Chawla N, et al. ‘The Hand on the Doorknob’: Visit agenda setting by complex patients and their primary care physicians. J Am Board Fam Med 2018;31(1):29–37; doi: 10.3122/jabfm.2018.01.170167 29330237 PMC5893137

[40] Robinson JD, Tate A, Heritage J. Agenda-setting revisited: When and how do primary-care physicians solicit patients' additional concerns? Patient Educ Couns 2016;99(5):718–723; doi: 10.1016/j.pec.2015.12.009 26733124

[41] Bensing JM, Tromp F, van Dulmen S, et al. Shifts in doctor-patient communication between 1986 and 2002: A study of videotaped general practice consultations with hypertension patients. BMC Fam Pract 2006;7:62; doi: 10.1186/1471-2296-7-62 17064407 PMC1630692

[42] Zimmerman DL, Min DJ, Davis-Collins A, et al.Treating patients as people: What do hospital patients want clinicians to know about them as a person? J Patient Exp 2020;7(2):270–274; doi: 10.1177/2374373519826244 32851151 PMC7427369

[43] Aelbrecht K, Rimondini M, Bensing J, et al. Quality of doctor-patient communication through the eyes of the patient: Variation according to the patient's educational level. Adv Health Sci Educ Theory Pract 2015;20(4):873–884; doi: 10.1007/s10459-014-9569-6 25428194

[44] Mazzi MA, Rimondini M, Boerma WG, et al. How patients would like to improve medical consultations: Insights from a multicentre European study. Patient Educ Couns 2016;99(1):51–60; doi: 10.1016/j.pec.2015.08.009 26337005

[45] Bensing J, Rimondini M, Visser A. What patients want. Patient Educ Couns 2013;90(3):287–290; doi: 10.1016/j.pec.2013.01.005 23395286

[46] van Vliet LM, Epstein AS. Current state of the art and science of patient-clinician communication in progressive disease: Patients' need to know and need to feel known. J Clin Oncol 2014;32(31):3474–3478; doi: 10.1200/JCO.2014.56.0425 25267758 PMC5569682

